# The decision-making process for unplanned admission to hospital unveiled in hospitalised older adults: a qualitative study

**DOI:** 10.1186/s12877-018-1013-y

**Published:** 2018-12-22

**Authors:** Maria Johanna van der Kluit, Geke J. Dijkstra, Sophia E. de Rooij

**Affiliations:** 1 University of Groningen, University Medical Center Groningen, University Center for Geriatric Medicine, Hanzeplein 1, 9700 RB Groningen, The Netherlands; 2 University of Groningen, University Medical Center Groningen, Department of Health Sciences, Applied Health Research, Hanzeplein 1, 9700 RB Groningen, The Netherlands

**Keywords:** Decision making, Older adults, Hospitalisation, Primary care, Patient perspective, Qualitative research, Grounded theory

## Abstract

**Background:**

The hazards of hospitalisation, and the growing demand for goal-oriented care and shared decision making, increasingly question whether hospitalisation always aligns with the preferences and needs of older adults. Although decision models are described comprehensively in the literature, little is understood about how the decision for hospitalisation is made in real life situations, especially under acute conditions. The aim of this qualitative study was to gain insight into how the decision to hospitalise was made from the perspective of the older patient who was unplanned admitted to hospital.

**Methods:**

Open interviews were conducted with 21 older hospitalised patients and/or their next of kin about the decision-making process leading to hospitalisation. Data were analysed according to the Constructivist Grounded Theory approach.

**Results:**

Although a period of complaints preceded the decision to unplanned hospitalisation, ranging from hours to years, the decision to hospitalise was always taken acutely. In all cases, there was an acute moment in which the home as a care environment was no longer considered adequate. This conclusion was based on a combination of factors including factors related to complaints, general practitioner and home environment. Three parties were involved in this assessment: the patient, his next of kin and the general practitioner. At the same time, a very positive value was attributed towards the hospital. Depending on the assessment of the home as care environment by the various parties, there were four routes to hospitalisation: referral, shared, demanding and bypassing.

**Conclusions:**

For all participants, the decision to hospitalisation was taken acutely, even if the problems evoking admission were not acute, but present for a longer period. Participants saw admission as inevitable, due to the negative perceptions of the care environment at home at that moment, combined with the positive expectations of hospital care. Advance care planning, nor shared decision making were rarely seen in these interviews. An ethical dilemma occurred when the next of kin consented to hospitalisation against the wishes of the patient. More attention for participation of older adults in decision making and their goals is recommended.

## Background

Hospitalisation is a major event for older adults. After hospitalisation at least 30% of older adults experience new or more functional decline [1, 2], often resulting in a temporary [3] or permanent discharge to nursing home [4] and 26% die within three months following hospitalisation [5]. Many older adults are not aware of this risk of functional decline and increased dependency after hospitalisation [6]. An increasing number of healthcare professionals have become aware of these hospital outcomes and this had led to adjustments in the hospitalisation process, such as introduction of the Comprehensive Geriatric Assessment and implementation of multidisciplinary teams or geriatric units [1, 7]. In addition, alternatives for in-hospital care were developed, such as outpatient management and the Hospital At Home care program [8]. Although these measures improved the outcomes for older hospitalised adults, still many older adults, especially the most vulnerable, die shortly after hospitalisation [9].

Both the hazards of hospitalisation and the growing demand for more goal-oriented care [10] and shared decision making [11] question whether care and treatment always align with the preferences and needs of older adults [12, 13]. As older adults are often hospitalised unplanned, time for shared decision making is often short or even lacking. In these situations, advance care planning, i.e. discussion about goals of care and making records of care preferences at an earlier stage [14] might have been a solution, although many older adults prefer to leave health decisions up to their doctor [15, 16].

Although models regarding decision making are described comprehensively in the literature [10, 14, 17], little is understood about how the decision for unplanned hospitalisation is made in real life situations, especially under unplanned conditions. The aim of this qualitative study was therefore to gain insight into how the decision to hospitalise was made from the perspective of the older hospitalised adult who was unplanned admitted to hospital.

## Methods

To gain insight into the decision-making process as experienced by older hospitalised adults, the Constructivist Grounded Theory approach [18] was used. This approach is useful in discovering the subjective experiences of participants, understanding social behaviours and describing processes [19].

### Dutch health care system

All Dutch residents are required to purchase basic health insurance, which covers the majority of essential medical care. The general practitioner (GP) refers the patient to a medical specialist and acts as a gatekeeper in making these referrals. During out-of-office hours, patients can contact the general practice centre. For severe medical emergencies, patients can visit the emergency room directly, or call 112 for an ambulance [20].

### Sample

Patients were recruited during their hospitalisation at the University Medical Centre Groningen (UMCG), a university teaching hospital in the Northern part of the Netherlands and at the Gelre Hospitals, a regional teaching hospital in the middle part of the Netherlands.

Inclusion criteria were: (1) unplanned medical hospitalisation expected for at least 48 h; (2) aged 70 years and older; (3) prefrail (1–2 points) or frail (> 3 points) according to the Fried-criteria as operationalized by Avila Funes [21], (4) being able to speak and understand Dutch; (5) not expected to die within the next 48 h; (6) informed consent to the interview and audio recording.

A theoretical sampling plan was used. Since acutely admitted older adults form a heterogeneous group, we aimed within eligible patients for maximum variation in age, pre-frail/frail patients, with and without cognitive impairment, living at home or in a nursing home, university hospital or regional hospital. According to the theoretical sampling strategy, some of these criteria were added during the process. For example, after having interviewed a patient admitted from a nursing home with a very passive role in the decision making, more patients from a nursing home or senior home were actively sought to check if this was a particular characteristic of nursing home residents. A regional hospital was visited to check whether the theory described so far only fitted university hospital patients, or if the same processes were seen in peripheral patients. We aimed to continue sampling until saturation was achieved, meaning the properties of our theoretical categories were saturated with data.

In total 26 interviews were conducted concerning 21 unique patients. Details of the sample are shown in Table 1. The characteristics and admission reason were asked to the patient or next of kin, when applicable. The admission term was retrieved from the hospital administration.

**Table 1:** Patient characteristics

	n
Gender	
Male	12
Female	9
Age (years)	
70–79	6
80–89	13
90–99	2
Admission term^a^	
< 5 days	3
5–10 days	11
> 10 days	9
Admission day interview^a^	
< 3 days	5
3–5 days	12
6–10 days	4
> 10 days	2
Fried score (points)	
1	1
2	3
3	4
4	11
5	2
Interview with	
Patient	15
Next of kin	3
Both	3
Number of interviews	
1	17
2	3
3	1
Living situation	
At home, alone without professional home care	5
At home, with partner without professional home care	4
At home, alone with professional home care	3
At home, with partner with professional home care	2
Senior home	3
Nursing home	4
Hospital	
UMCG	19
Gelre	2
Admission due to^a^	
Dyspnoea	10
Fall	3
Constipation	3
Swollen leg	2
General malaise	2
Abdominal pain	1
Diarrhoea	1
Urinary tract infection	1

^a^Some patients were interviewed during two different admissions

#### Procedure

The researchers were not involved in de patient care, nor had contact before admission with the patients. After establishing inclusion criteria by the staff nurse, eligible patients were given an informational letter and were approached by the interviewer (MJvdK) for further information about the procedure and to obtain informed consent during their hospitalisation. In case a patient had an impaired consciousness or cognition, the next of kin was approached for an interview and informed consent of the next of kin was obtained.

### Data collection

Open interviews were conducted during hospitalisation by the first author (MJvdK) between June 2016 and August 2017. The opening question was: In your opinion, what was the reason for your hospitalisation? Development of the interviews was dependent on each participant discussing topics that were relevant to them. A topic-list, adapted during the process, was used as a flexible guide during all interviews. Examples of topics were: when was the decision made, who were involved, what was the decisive reason, view on hospital. Interviews took place in the patient room or, when the patient shared a room, in a family or examination room on the ward. Patients were explicitly given the opportunity to finish the interview, for example in case of fatigue, this was also checked by the interviewer. Three interviews with a next of kin were conducted by telephone, since it was not possible for them to come to the hospital. The interviews took 15 to 60 min and were audio-recorded and transcribed verbatim.

### Data analysis

Characteristic for grounded theory is the iterative process and constant comparison [18, 19, 22]. Therefore, data collection and analysis occurred simultaneously. Coding was done inductively according to the following steps: initial coding, focused coding, axial coding and theoretical coding [18, 22].

Initial coding took place as soon as possible after the interview and transcription. With initial coding, the researcher remained close to the data, and coded open and spontaneously. During focused coding the most significant or frequent initial codes were selected in order to code larger amounts of data. Codes were more conceptual. With axial coding the various dimensions and characteristics of the identified concepts were described under which conditions, actions/interactions and consequences they occurred.

With theoretical coding the relationships between the categories were described, to be integrated in theory forming.

In all stages constant comparison was used: new codes, theories and relationships were compared with each other and with previous data to identify similarities and differences in the data, define and sharpen concepts and to verify the constructed theory and memos were written during the entire process [18, 22]. For example, when new codes emerged, it was checked whether these were applicable for previous cases. Every time a new category was constructed, it was checked whether all the previous cases fitted in the categories and a description was made, based on the data. After a new interview, it was checked whether the new case fitted in the existing categories, or whether a new description or a new category was needed, until all categories were saturated.

The first two transcripts were coded by the first (MJvdK) and second author (GJD) independently and then compared. The subsequent transcripts were coded by the first author alone, but also read by the second author. Major codes and memos were discussed together.

Data analysis and organization was supported by the use of Atlast.ti Version 5.2.18.

## Results

**Fig. 1:**
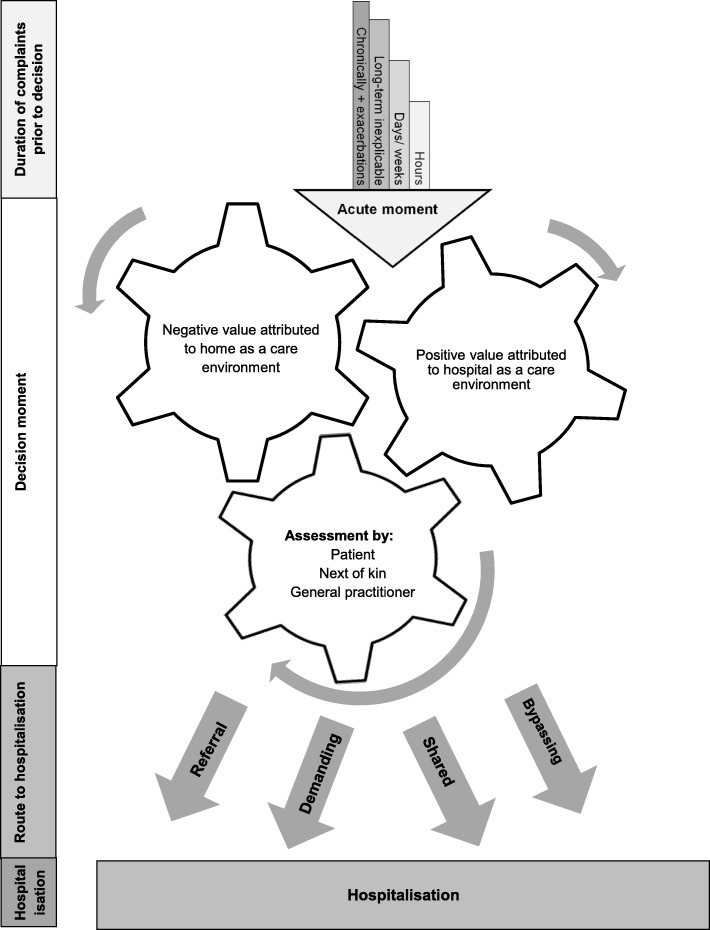
Graphical summary of the four stages of the decision making process. The arrow in the top of the figure represents the period of complaints preceding the decision to hospitalisation ranging from hours to years and ends in an acute moment for all cases, when the decision moment takes place. In the decision moment, the home situation as a care environment was no longer considered adequate. At the same time, a very positive value was attributed to the hospital. Three parties were involved in this assessment: the patient, his next of kin and the general practitioner. Depending on the assessment of the home situation as a care environment by the three parties, there were four routes to hospitalisation: Referral, Demanding, Shared, Bypassing. Only the category “Shared” was not saturated

The decision process was divided into four stages: (1) duration of complaints prior to decision to hospitalisation; (2) the decision moment, in which an assessment was made of the current care situation at home and the expectations of hospital care. This resulted in (3) various routes to hospitalisation and finally was (4) reflected on the decision made. The results are summarised graphically in Fig. 1.

### Duration of complaints prior to decision to hospitalise

A period of illness or complaints preceded the decision to hospitalise, ranging from hours to years. This period can be divided into four categories:

#### Hours

The first category concerned acute, spontaneous problems, for example acute cardiac complaints in previously (reasonably) healthy older adults.

#### Days/weeks

In the second category, patients had complaints in the previous days or weeks, such as constipation, recurrent episodes of shortness of breath, or a combination of several inexplicable complaints that did not improve, despite treatment by the general practitioner (GP).

#### Long-term inexplicable

The third category consisted of patients with complaints in the months, and sometimes up to several years, prior. These concerned long-term, inexplicable, complaints interfering with daily functioning (walking, activities of daily living, cognition, relationships), for which no help was sought, the patient felt not understood, care providers were unable to resolve complaints, or help was avoided. In such cases, an acute situation such as a fall or infection, was the straw that broke the camel’s back. The motivation for hospitalisation was not the incident, but the precursory complex situation.

#### Chronically with exacerbations

The fourth category consisted of people with a chronic illness who were regularly re-admitted due to an exacerbation.

### Decision moment

The decision leading to hospitalisation was taken acutely in almost all cases, meaning that prior to the moment of admission the patient had not had thoughts nor conversations with care providers or next of kin about the desirability of hospitalisation. In contrast, two interviewees, living in a senior home and nursing home respectively, had previously discussed with their doctor and family in which situations they would or would not want to be hospitalised.

In all cases the negative value that was attributed to the current care situation at home, was the reason for admission. Three parties, with varying degrees of influence, were involved in that process: the patient, the GP and the next of kin.

#### The negative value that was attributed to home as a care environment

The patient’s environment at the decision moment was considered inadequate by all interviewees. The environment could be at home with help of the GP, combined or not with homecare and/or informal care, or a nursing home. The factors that led to the decision to acute hospitalisation were: (1) Reaching the limit of the duration of the complaints; (2) intensity and progression of the complaints; (3) Competences and capabilities of the GP; (4) lack of confidence in the GP; (5) functional decline and (6) capacity of the informal caregiver.

In addition, several factors contributed to a patient’s feeling of a less pleasant home situation, for example not finding peace at home due to an inconveniencing housemate or the low quality of service in the nursing home that was experienced due to a shortage of painkillers needed or the poor quality of the food provided. These were not decisive reasons for hospitalisation, but played a role in the evaluation for the interviewee.

#### Duration of the complaints is too long

For some patients the limit to the duration of the complaints was reached. This could be indicated in several ways: (1) the patient suffered considerably from persistent complaints and could not bear this any longer; (2) an acute situation had arisen with long-standing problems that made the patient or next of kin feel he reached his limit; or (3) the GP indicated that the symptoms lasted too long.

*“But because he had fallen (…) then my mother and the housekeeper, yes they had also explained the situation to us. That he had stood with a raised hand to my mother that weekend and that he had been very angry and you name it. And that she also said like: I cannot handle this any longer. So then she phoned* [to the GP] *that something had to change.” (Daughter P18).*

#### The progression and intensity of the complaints

For other patients, duration of the complaints was not so much the problem, but the increased intensity, which led to anxiety among patient, next of kin or GP. Sometimes the complaints were so severe that the patient or informal carer expected the patient would certainly die if he had to stay at home.

*“At the very end of the day, it was so far that my wife no longer trusted herself with the situation.” (P04).*

#### Competences and capabilities of the (locum) GP

A number of interviewees discussed that the GP did not know how to deal with the situation, for example because the GP was a locum not knowing the patient, or had a lack of diagnostic resources available at home or because the case was beyond the scope of the GP.

*“They want to investigate of course what, exactly, the problem is. And those diagnostic procedures, you cannot do that in, uh, such a nursing home”. (Son P17).*

#### Lack of confidence in GP

Another factor was a lack of confidence in skills of their own GP. Interviewees indicated that the GP provided advice or interventions which were useless, according to the interviewee, or did too little to address the symptoms, waited too long to refer or did not know what to do.

*“Yes, I had diarrhoea several times. (…) I then started to vomit. Well, the doctor did not really react, he said at some point: “She has had it before, it will be over again.” But now it was so bad and my back, too, and I was crazy from it, really. I couldn’t bear the pain any longer. That’s why I was admitted here.” (P13).*

#### Functional decline

A number of patients had difficulties in activities of daily living such as self-care, walking, getting into bed and eating and drinking because of illness.

Patient: *“Since if I would be at home now, everyone had to show up. One had to do this, the other had to do that. And now you’re being taken care of and all sorts of things.”* Interviewer: “*Yes, because you could no longer take care of yourself at home?”* Patient: “*No. In the condition I was, no.” (P06).*

#### Caregiver burden

Some next of kin indicated that having a seriously ill loved one at home caused much worry and stress. In other cases the care was considered too much or too time-consuming for the informal caregiver.

*“The worries I have at home, when he is ill, have given me so much stress over the years. I cannot really handle that anymore. And when he is in Groningen then I am reassured that he is, uh, that there are doctors who are constantly watching him, that there are nurses with him if anything happens.” (Spouse P04).*

#### Positive value attributed to the hospital as a care environment

While the home situation was considered inadequate, the interviewees had a very positive view on the hospital. This was summarised by one patient as: “*The hospital is the best place to be when you are unhealthy” (P09).* The in-hospital service was highly valued: staff were friendly and helpful, meals were good, and the support was perceived as better than in the nursing home for those who lived there.

Advantages of hospitalisation according to the interviewees were assistance and care offered, thorough diagnostic procedures, possibility to intervene and better control and treatment opportunities. In addition, patients often recovered due to treatment in the hospital, this could be an experience during the current hospital admission or after a previous admission and the recoveries ranged from “a tiny little bit” to a “total reborn”. Or the hospitalisation made the patient survive.

The hospital also gave hope. Hope to improve, to relieve symptoms, to survive or to achieve personal goals.

*“So you always hope that they can do something for you. I mean, you try to get your benefits out of that. Yes, I mean, you depend on those doctors. So if they can do something for you, that everything becomes easier, your life becomes easier, your breathing and uh, those painful bursts of coughing fits and you name it, that it stops. So you have that hope. And if it works, that that … Well … Sometimes it does and sometimes it doesn’t, otherwise I would not have had those four hospitalisations.” (P07).*

Finally, hospitalisation also provided rest to the informal caregiver regarding daily care, worries and stress.

### Route to hospitalisation

The admission decision followed four routes: the GP refers to the hospital, the referral is demanded by the patient, the referral takes place after shared decision making between the patient or next of kin and the GP, the GP is bypassed by patient and/or next of kin.

#### The GP refers the patient to the hospital

The GP referred the patient to the emergency department and this decision originated completely with the GP, sometimes in consultation with the medical specialist. The patient did not have a say in these cases. Sometimes this was not possible due to the urgency, or the patient was too ill to participate actively in decision-making. In other cases, the patient indicated that he did not want to have a say. In all these cases there was great confidence in the referrer. Patients often had the idea that “if the doctor says: if it must be, then so be it”.

*“And uh, then I called the emergency doctor, yeah, called in the middle of the night and they arrived very quickly, within an hour. (…) Two doctors came and they saw me and they said to me: “Sir, but sir, you must go to the hospital immediately.” They then called the ambulance. The ambulance arrived within half an hour. And they brought me to the UMCG.” (P03).*

#### The referral is demanded by the patient

In other situations, the patient, sometimes with help of his next of kin, demands to the GP a referral to the hospital. In these cases the limit of the duration of the complaints is reached earlier for the patient than for the GP, sometimes combined with a lack of confidence in the GP.

Patient: *“In the end, I really hammered home. I said, I don’t want this any longer.”* Interviewer: *“Yes. So then you really…”* Patient: *“I really blew the whistle, yes.”* Interviewer: *“Yes, and what was the point that you thought of well, this can’t go on like this?”* Patient: *“Well, I was fed up. Vomiting and with defecation, oh man, I couldn’t bear it any longer. (…) I said to that doctor.. I said I can’t bear this any longer, I want to go to the hospital.” (P11).*

#### The referral takes place after shared decision making between the next of kin and the GP

In one case, a GP offered a choice to a patient’s son and asked whether he wanted to send his father to the hospital or preferred palliative care at home. However, this unexpected choice was experienced as particularly difficult due to the great responsibility the patient’s son felt by this choice.

*“Look, the doctor may come, or a locum, and say: “yes what do you want? That man has been so sick already, does he have to go to hospital now, in his condition?” Look, I won’t have that. Because I still would like him to…, that he still gets a chance, to be looked at. (…) I find that a very difficult choice, because then the trouble is dropped at my door. And then I have to let my father die, actually. Look, I think that’s a bit of a difficult choice.” (Son P14).*

#### The GP is bypassed in the decision-making

By calling an ambulance or intentionally consulting the GP at the weekend to arrange a visit by a locum GP, the own GP was bypassed. This was done because the patient and sometimes his next of kin had no longer confidence in the GP and sought another route to find help for the complaints.

Patient: *“I say, “You know what, we’re going to call the ambulance and then they come, something has to be done.” Whatever happens. (…)”* Interviewer: *“And uh, what was the reason, you thought: I have to do this myself and not by the GP?”* Patient: “*That makes no sense, because that GP won’t do anything.” (P05).*

### Reflection on decision to hospitalise

All interviewed patients and next of kin were satisfied with the decision to hospitalise. This was because of the mentioned advantages of hospitalisation compared with the current home situation. For many the decision to hospitalise was self-evident and the only option. Often this was formulated along the lines of “there was no choice”. Moreover, there was often great confidence in the decision that the GP had taken for the patient.

*“Those doctors know about how and what. And so I have to surrender. And so I have at that moment, at that moment I have nothing to say, because it is all for my own good. And then I’m done with that. (…) If I say: “Well, I do not want to be hospitalised, I go home.” Yes, I’m hurting only myself. So automatically you agree and I think that is logical.” (P07).*

In a few cases, an interviewed adult child indicated that he had made the decision to hospitalise, but perhaps against the wishes of his parent. The choice for hospital admission was then made by the child because he assumed that the patient was not capable of making the decision himself and because the child wanted everything to be done to keep his parent alive.

*“Look, then, uhm, he sometimes becomes afraid of the hospital. Then I think: not again!, then he must go again. That is sometimes difficult. In this case also, when we went to the hospital, he said, I want to go home, because I do not feel like it. (…) He does not want so much anymore. I think, to be blunt, if it ends, he will not be very sad about it, actually. I have sometimes…, he is actually a bit finished with it. Do you understand what I mean? Look, we* [children] *still want everything, but… He has actually a bit, since my mother passed away, actually is uhm, his part, when my mother died, we actually said to each other, my father also died a bit.” (Son P14).*

## Discussion

In this study the decision-making process for hospitalisation as experienced by older hospitalised patients and their next of kin was investigated. For all participants, the decision to hospitalisation was taken acutely, even if the problems evoking admission were not acute, but present for a longer period. Participants saw admission as inevitable, there was no choice. This was caused by the assessment by the patient, his next of kin and/or GP that the care provided at home or by the nursing home was insufficient, combined with the expectation that the best care was provided in the hospital with the greatest chance of cure or comfort.

As far as we know, this is one of the few studies into the decision-making process of unplanned hospital admission in which is spoken with a broad group of older adults themselves during their hospitalisation. Other studies were observational [23], or after discharge and only concerned patients with home care [24], or a vignette study with heart failure patients [25], targeted care providers [26, 27], were quantitative and only focused on functional decline [28], or only focused on nursing home residents [29–31].

The patients and next of kin interviewed in this study all considered hospitalisation as inevitable. This was also found in previous research [25, 31, 32]. A possible explanation for this is given by Lynn et al. [33] who reason that since physicians and patients use routine behaviour, especially under time pressure, decision making moments are often not recognized. Therefore, unplanned hospital admission is often not recognized as a moment of decision, but is considered as the default option in case of an acute emerging illness and people do not feel they have a choice [33]. “Doing nothing”, in this case receiving treatment at home, is by some patients not considered as an option and therefore suggests there is no decision to be made [34].

Many interviewees in this study had a passive role in the decision-making process and were very confident in the solution chosen by their care providers. Likewise, earlier studies also revealed a group of older adults with great confidence in physicians and who were satisfied with an obviously passive role in decision-making [34–36]. It is a well-known phenomenon that people with a lot of trust in health care providers, which is more common among older adults, often prefer a passive role in decision-making [15, 37].

However, this passive role not always reflects a conscious choice, but can also be seen as resignation. In previous research among nursing home residents who were referred to the emergency room, residents reported that their opinion had never been asked, but many were not dissatisfied with this although some would have liked it differently [31].

In addition, this research revealed that people were sometimes too ill to be able to make a decision themselves. This was also seen in previous research [34, 35, 38]. To hand over decisions can as well be related to the sick role [16].

All interviewees in this study were satisfied with the decision to hospitalise. This could be explained by hindsight bias, people look back on the decision afterwards and consider this in the light of the final outcome [39]. For some people, the outcome was that they recovered by treatment in hospital, for others that they received intensive care, which can also confirm that the decision to admit was the right choice [32].

As in our study, the negative perception of home as a care environment was shown in other studies. For instance, bypassing the patient’s own GP because of dissatisfaction has been previously reported in research among GP-led urgent care centres and emergency rooms [40, 41]. Lack of confidence, which is more often reported in nursing homes, where family has often too little trust in the staff, is also a motive to go to hospital [29, 42, 43].

This study showed various examples of situations in which the GP had insufficient knowledge due to unfamiliarity with the patient. In previous research GPs also indicated that if they missed necessary background information, this uncertainty led to (inappropriate) referrals to hospital [27, 44].

Functional decline also contributed, as evidenced in earlier research, to the reason for visiting the emergency room by older adults [28]. Finally, earlier research also showed that older adults preferred hospitalisation to relieve their families [45, 46], or families insisted on hospitalisation because of the concerns about their loved one at home [24].

The positive perception of the hospital is also reflected in various studies: symptoms can be investigated [25], help is quickly and always available [24, 45, 46], the treatment is perceived as better than at home [24, 29, 47], the patient makes better progress [45, 46, 48], the survival chances are perceived to be higher [25, 45], people feel more secure through closer monitoring [24, 31, 45, 46], patients do not have to burden their families [45, 46] and the meals are better [24].

Interesting is the expressed need for hope. Although a few patients returned to the hospital often without considerable benefit, this readmission still had added value for them, since they received hope. While an exacerbation can contribute to a feeling of hopelessness [49], control of symptoms, belief in achieving remission, setting goals and receiving information from professionals engender hope [50]. Apparently, for some patients and next of kin, the hospital is the best place or last resource to receive this hope, and even without any real benefit, they have at least tried.

The cases concerning acute onset of symptoms necessitating an urgent decision were rare in this study. In most cases, the symptoms have already existed for days to weeks or even chronically. Especially in the latter case, it is remarkable that the patient’s wishes regarding hospitalisation and treatment were rarely discussed at an earlier stage. Advance care planning was rarely seen in these interviews, except for two nursing home residents. Literature reviews showed that studies into the effect of *do-not-hospitalise orders* were all performed among nursing home residents [14] and that advance care planning had taken place only in a minority of non-sudden deaths [51]. The fact that advance care planning was rarely seen in this study, could be caused by patients having a do-not-hospitalise order that was followed, thus keeping them out of the hospital. Nevertheless, it is remarkable that many of the interviewed chronically ill community dwelling older adults had never discussed their wishes regarding hospitalisation. An explanation may be that patients in need of palliative or supportive care are often not recognized by GPs, which makes the initiation of advance care planning more difficult. This is especially true for patients with end-stage organ failure and dementia [52, 53].

Many interviewed older adults in this study had a very passive role; nobody had ever presented them with a choice regarding hospitalisation. Only one choice was presented to one patient’s son who found this choice too difficult. Older adults have been brought up more often with the idea that the ideal patient is obedient and passive and expressing one’s own preferences is sometimes even considered selfish [54]. But the fact that older adults do not participate, or participate less frequently, in decision-making, does not mean this desire does not exist. It is present, but is determined very individually [35, 38, 54]. Often a discrepancy exists between the actual and the preferred role [35, 38].

Another explanation as to why many patients did not participate in the decision and often did not want to, is that many older adults are not focused on whether interventions are useful or not, but more on which outcomes they find important [55, 56]. So the question should not be: do you want to be hospitalised or not, but what do you want to achieve? It is then up to the healthcare provider to decide whether hospitalisation could help to achieve that goal.

And hospital outcomes are not always better than treatment at home or in a nursing home. In hospital treatment can sometimes even be worse [8, 57–60], although this is difficult for many patients to understand [45].

Interviewed patients were very confident that their doctor and next of kin would make the right decisions for them, but remarkably they did not share their preferences with them. This is seen more often in the literature [56, 61, 62]. Kuluski et al. [63], showed that goals of patients, family members and GPs often did not align, especially in cases of unstable health and high complexity. Absence of knowledge of the patient’s goals has several disadvantages, such as avoidable or unnecessary hospitalisations of nursing home residents [29, 64], or placing stress on family members whether they take the right decision if they find themselves in an acute situation [61].

This could explain the overchoice experienced by an interviewed son. He really wanted to make the right decision for his father, but his father did not want to talk about this subject. The son wanted to seize every available chance for survival, even though he knew these prospects were very limited. At the same time, he felt that staying alive was not his father’s priority. The stress caused by the unexpected question of a locum GP about whether or not to hospitalise, is very understandable. According to Lynn et al. [33], family members are often afraid of later regretting their decision not to have seized every chance for survival. Moreover, people follow rules they consider appropriate to the situation in which they find themselves. If a son feels that a good son must do everything to prolong his father’s life, he does so, regardless of prognoses [33].

Literature about decisions made by family members of nursing home residents showed families struggling with these dilemmas. Family members want to do well, see themselves as the patient’s advocate and find quality of life of the resident important. Not wanting treatments that give discomfort with little benefit, however, families often suffer from prognostic uncertainty [48, 64]. Sometimes the family did not want a *do-not-hospitalise order* until they were certain the resident was near the end of life [64].

Two adult children in this study indicated that they might have sent their parent to hospital against his wishes. Overruling patients by family members to go to hospital is not unique [25, 31, 48]. Hopp et al. [25], describe several communication styles between next of kin and patient. These vary from influential to directive. In the last style the next of kin perceives a need to carry out his desired course of action without consideration of the patient’s preferences. It is also known that if people have to decide for others, they opt for an active treatment more often than for themselves [65], and they overestimate the wish for treatment [62].

### Strengths and limitations

The qualitative method gave a rich insight into the perspective of the patient and his or her next of kin when it comes to the entire decision-making process leading to hospitalisation. However, this method also had some limitations. Firstly, the interview took place after the patient had been admitted for a number of days. As a result, interviewees may have forgotten details of the admission process and the patient may have assessed the situation differently because he was hospitalised.

Furthermore, we did not succeed to achieve saturation for one category, namely patients who made the decision to hospitalisation by shared decision making, since this appeared a rare phenomenon and we could not find other patients in this category. With only one case, we have not discovered all aspects of this category, in which circumstances this took place and whether one (negative) experience is representative for other cases of shared decision making.

Although, as shown in the discussion, many factors involved in the decision making process are seen in the literature worldwide, communication between patients, next of kin and doctors differ among cultures, as do healthcare systems and access to care. Therefore, we are unsure to which extent the results are transferable to other contexts.

## Conclusions

The decision to hospitalise in older adults was often taken acutely, even when the care needs were not acute, but present for a longer period. Admission was seen as inevitable by patients and their next of kin. This might occur due to the negative perceptions of the care environment at home at that moment, combined with the positive expectations of hospital care. Participation in decision-making by the older adult himself and advance care planning were scarce. Furthermore, dilemmas were revealed with sometimes conflicting interests.

Future research should focus on the desires of older adults regarding active or passive participation in the decision making process and on their goals regarding the outcome of hospitalisation.

## Abbreviations

GP: General practitioner

## References

[1] Lafont C, Gérard S, Voisin T, Pahor M, Vellas B (2011). Reducing "iatrogenic disability" in the hospitalized frail elderly. J Nutr Health Aging.

[2] Buurman BM, Hoogerduijn JG, de Haan RJ, Abu-Hanna A, Lagaay AM, Verhaar HJ (2011). Geriatric conditions in acutely hospitalized older patients: prevalence and one-year survival and functional decline. PLoS One.

[3] Liu SK, Montgomery J, Yan Y, Mecchella JM, Bartels SJ, Masutani R (2016). Association between hospital admission risk profile score and skilled nursing or acute rehabilitation facility discharges in hospitalized older adults. J Am Geriatr Soc.

[4] Portegijs E, Buurman BM, Essink-Bot M, Zinderman AH, De Rooij SE (2012). Failure to regain function at 3 months after acute hospital admission predicts institutionalization within 12 months in older patients. J Am Med Dir Assoc.

[5] de Rooij SE, Buurman BM, Korevaar JC, Van Munster BC, Schuurmans MJ, Laqaaij AM (2007). Co-morbidity in acutely hospitalised older patients as a risk factor for death in hospital or within 3 months after discharge. Ned Tijdschr Geneeskd.

[6] Boltz M, Capezuti E, Shabbat N, Hall K (2010). Going home better not worse: older adults' views on physical function during hospitalization. Int J Nurs Pract.

[7] Ellis G, Whitehead MA, Robinson D, O'Neill D, Langhorne P (2011). Comprehensive geriatric assessment for older adults admitted to hospital: meta-analysis of randomised controlled trials. BMJ.

[8] Conley J, O'Brien CW, Leff BA, Bolen S, Zulman D (2016). Alternative strategies to inpatient hospitalization for acute medical conditions: a systematic review. JAMA Intern Med.

[9] van Rijn M, Buurman BM, MacNeil-Vroomen JL, Suijker JJ, ter Riet G, Moll van Charante EP (2016). Changes in the in-hospital mortality and 30-day post-discharge mortality in acutely admitted older patients: retrospective observational study. Age Ageing.

[10] Reuben DB, Tinetti ME (2012). Goal-oriented patient care--an alternative health outcomes paradigm. N Engl J Med.

[11] Chewning B, Bylund CL, Shah B, Arora NK, Gueguen JA, Makoul G (2012). Patient preferences for shared decisions: a systematic review. Patient Educ Couns.

[12] Schoon Y, van Iersel M, Jacobsen D, Smit JWA, de Boer M, Olde Rikkert MGM (2013). Improved hospital care for elderly patients: guidance on vulnerability and goal assessment. Ned Tijdschr Geneeskd.

[13] Mulley A, Trimble C, Elwyn G (2012). Stop the silent misdiagnosis: patients' preferences matter. BMJ.

[14] Brinkman Stoppelenburg A, Rietjens JAC, van der Heide A (2014). The effects of advance care planning on end-of-life care: a systematic review. Palliat Med.

[15] Chawla N, Arora KN (2013). Why do some patients prefer to leave decisions up to the doctor: lack of self-efficacy or a matter of trust?. J Cancer Surviv.

[16] Say R, Murtagh M, Thomson R (2006). Patients' preference for involvement in medical decision making: a narrative review. Patient Educ Couns.

[17] Elwyn G, Frosch D, Thomson R, Joseph-Williams N, Lloyd A, Kinnersley P (2012). Shared decision making: a model for clinical practice. J Gen Intern Med.

[18] Charmaz K (2014). Constructing grounded theory.

[19] Higginbottom G, Lauridsen E (2014). The roots and development of constructivist grounded theory. Nurse Res.

[20] European Observatory on Health Systems and Policies. The Health systems and policy monitor. Available at: https://www.hspm.org/mainpage.aspx. Accessed 20 Nov 2018.

[21] Avila Funes JA, Helmer C, Amieva H, Barberger Gateau P, Le Goff M, Ritchie K (2008). Frailty among community-dwelling elderly people in France: the three-city study. J Gerontol A Biol Sci Med Sci.

[22] Giles TM, de Lacey S, Muir-Cochrane E (2016). Coding, constant comparisons, and Core categories: a worked example for novice constructivist grounded theorists. ANS Adv Nurs Sci.

[23] Dyrstad DN, Laugaland K, Storm M (2015). An observational study of older patients' participation in hospital admission and discharge--exploring patient and next of kin perspectives. J Clin Nurs.

[24] Hallgren J, Ernsth Bravell M, Dahl Aslan KA, Josephson I (2015). In hospital we trust: experiences of older peoples' decision to seek hospital care. Geriatr Nurs.

[25] Hopp FP, Marsack C, Camp JK, Thomas S (2014). Go to the hospital or stay at home? A qualitative study of expected hospital decision making among older African Americans with advanced heart failure. J Gerontol Soc Work.

[26] Skirbekk H, Nortvedt P (2014). Inadequate treatment for elderly patients: professional norms and tight budgets could cause "ageism" in hospitals. Health Care Anal.

[27] Hammond CL, Pinnington LL, Phillips MF (2009). A qualitative examination of inappropriate hospital admissions and lengths of stay. BMC Health Serv Res.

[28] Wilber ST, Blanda M, Gerson LW (2006). Does functional decline prompt emergency department visits and admission in older patients?. Acad Emerg Med.

[29] Arendts G, Quine S, Howard K (2013). Decision to transfer to an emergency department from residential aged care: a systematic review of qualitative research. Geriatr Gerontol Int.

[30] Dwyer R, Stoelwinder J, Gabbe B, Lowthian J (2015). Unplanned transfer to emergency departments for frail elderly residents of aged care facilities: a review of patient and organizational factors. J Am Med Dir Assoc.

[31] Arendts G, Popescu A, Howting D, Quine S, Howard K (2015). 'They never talked to me about… ': perspectives on aged care resident transfer to emergency departments. Australas J Ageing.

[32] Themessl Huber M, Hubbard G, Munro P (2007). Frail older people's experiences and use of health and social care services. J Nurs Manag.

[33] Lynn J, Arkes HR, Stevens M, Cohn F, Koenig B, Fox E (2000). Rethinking fundamental assumptions: SUPPORT's implications for future reform. Study to Understand Prognoses and Preferences and Risks of Treatment. J Am Geriatr Soc.

[34] Joseph Williams N, Elwyn G, Edwards A (2014). Knowledge is not power for patients: a systematic review and thematic synthesis of patient-reported barriers and facilitators to shared decision making. Patient Educ Couns.

[35] Ekdahl AW, Andersson L, Friedrichsen M (2010). "they do what they think is the best for me." frail elderly patients' preferences for participation in their care during hospitalization. Patient Educ Couns.

[36] Dyrstad DN, Testad I, Storm M (2015). Older patients' participation in hospital admissions through the emergency department: an interview study of healthcare professionals. BMC Health Serv Res.

[37] Kraetschmer N, Sharpe N, Urowitz S, Deber RB (2004). How does trust affect patient preferences for participation in decision-making?. Health Expect.

[38] Ekdahl AW, Andersson L, Wiréhn A, Friedrichsen M (2011). Are elderly people with co-morbidities involved adequately in medical decision making when hospitalised? A cross-sectional survey. BMC Geriatr.

[39] Redelmeier DA, Rozin P, Kahneman D (1993). Understanding patients' decisions. Cognitive and emotional perspectives. JAMA.

[40] Diserens L, Egli L, Fustinoni S, Santos Eggimann B, Staeger P, Hugli O (2015). Emergency department visits for non-life-threatening conditions: evolution over 13 years in a Swiss urban teaching hospital. Swiss Med Wkly.

[41] Greenfield G, Ignatowicz A, Gnani S, Bucktowonsing M, Ladbrooke T, Millington H (2016). Staff perceptions on patient motives for attending GP-led urgent care centres in London: a qualitative study. BMJ Open.

[42] Stephens C, Halifax E, Bui N, Lee SJ, Harrington C, Shim J (2015). Provider perspectives on the influence of family on nursing home resident transfers to the emergency department: crises at the end of life. Curr Gerontol Geriatr Res.

[43] Trahan LM, Spiers JA, Cummings GG (2016). Decisions to transfer nursing home residents to emergency departments: a scoping review of contributing factors and staff perspectives. J Am Med Dir Assoc.

[44] McDermott C, Coppin R, Little P, Leydon G (2012). Hospital admissions from nursing homes: a qualitative study of GP decision making. Br J Gen Pract.

[45] Fried TR, van Doorn C, Tinetti ME, Drickamer MA (1998). Older persons' preferences for site of treatment in acute illness. J Gen Intern Med.

[46] Fried TR, van Doorn C, O'Leary JR, Tinetti ME, Drickamer MA (2000). Older person's preferences for home vs hospital care in the treatment of acute illness. Arch Intern Med.

[47] Low JA, Chan DKY, Hung WT, Chye R (2003). Treatment of recurrent aspiration pneumonia in end-stage dementia: preferences and choices of a group of elderly nursing home residents. Intern Med J.

[48] Abrahamson K, Bernard B, Magnabosco L, Nazir A, Unroe KT (2016). The experiences of family members in the nursing home to hospital transfer decision. BMC Geriatr.

[49] Rosa F, Bagnasco A, Ghirotto L, Rocco G, Catania G, Aleo G (2018). Experiences of older people following an acute exacerbation of chronic obstructive pulmonary disease: a phenomenological study. J Clin Nurs.

[50] Broadhurst K, Harrington A (2015). A mixed method thematic review: the importance of hope to the dying patient. J Adv Nurs.

[51] Glaudemans JJ, Moll van Charante EP, Willems DL (2015). Advance care planning in primary care, only for severely ill patients? A structured review. Fam Pract.

[52] De Vleminck A, Pardon K, Beernaert K, Deschepper R, Houttekier D, Van Audenhove C (2014). Barriers to advance care planning in cancer, heart failure and dementia patients: a focus group study on general practitioners' views and experiences. PLoS One.

[53] Thoonsen B, Groot M, Verhagen S, van Weel C, Vissers K, Engels Y (2016). Timely identification of palliative patients and anticipatory care planning by GPs: practical application of tools and a training programme. BMC Palliat Care.

[54] Foss C (2011). Elders and patient participation revisited - a discourse analytic approach to older persons' reflections on patient participation. J Clin Nurs.

[55] Rosenfeld KE, Wenger NS, Kagawa Singer M (2000). End-of-life decision making: a qualitative study of elderly individuals. J Gen Intern Med.

[56] Romo RD, Allison TA, Smith AK (2017). Sense of control in end-of-life decision-making. J Am Geriatr Soc.

[57] Boockvar KS, Gruber Baldini AL, Burton L, Zimmerman S, May C, Magaziner J (2005). Outcomes of infection in nursing home residents with and without early hospital transfer. J Am Geriatr Soc.

[58] Dosa D (2005). Should I hospitalize my resident with nursing home-acquired pneumonia?. J Am Med Dir Assoc.

[59] Covinsky K, Pierluissi E, Johnston CB (2011). Hospitalization-associated disability: "she was probably able to ambulate, but I'm not sure". JAMA.

[60] Dwyer R, Gabbe B, Stoelwinder JU, Lowthian J (2014). A systematic review of outcomes following emergency transfer to hospital for residents of aged care facilities. Age Ageing.

[61] Bollig G, Gjengedal E, Rosland JH (2016). They know!-do they? A qualitative study of residents and relatives views on advance care planning, end-of-life care, and decision-making in nursing homes. Palliat Med.

[62] Fagerlin A, Ditto PH, Danks JH, Houts RM, Smucker WD (2001). Projection in surrogate decisions about life-sustaining medical treatments. Health Psychol.

[63] Kuluski K, Gill A, Naganathan G, Upshur R, Jaakkimainen RL, Wodchis WP (2013). A qualitative descriptive study on the alignment of care goals between older persons with multi-morbidities, their family physicians and informal caregivers. BMC Fam Pract.

[64] Mann E, Goff SL, Colon-Cartagena W, Bellantonio S, Rothberg MB (2013). Do-not-hospitalize orders for individuals with advanced dementia: healthcare proxies' perspectives. J Am Geriatr Soc.

[65] Zikmund-Fisher BJ, Sarr B, Fagerlin A, Ubel PA (2006). A matter of perspective: choosing for others differs from choosing for yourself in making treatment decisions. J Gen Intern Med.

